# Asparaginase-specific basophil recognition and activation predict Asparaginase hypersensitivity in mice

**DOI:** 10.3389/fimmu.2024.1392099

**Published:** 2024-04-15

**Authors:** Sanjay Rathod, Keito Hoshitsuki, Yin Zhu, Manda Ramsey, Christian A. Fernandez

**Affiliations:** Center for Pharmacogenetics and Department of Pharmaceutical Sciences, University of Pittsburgh, Pittsburgh, PA, United States

**Keywords:** Asparaginase, anaphylaxis, drug hypersensitivity, CD200R3, basophil activation test (BAT), immune complex

## Abstract

**Background:**

Asparaginase (ASNase) is a crucial part of acute leukemia treatment, but immune responses to the agent can reduce its effectiveness and increase the risk of relapse. Currently, no reliable and validated biomarker predicts ASNase-induced hypersensitivity reactions during therapy. We aimed to identify predictive biomarkers and determine immune cells responsible for anaphylaxis using a murine model of ASNase hypersensitivity.

**Methods:**

Our preclinical study uses a murine model to investigate predictive biomarkers of ASNase anaphylaxis, including anti-ASNase antibody responses, immune complex (IC) levels, ASNase-specific binding to leukocytes or basophils, and basophil activation.

**Results:**

Our results indicate that mice immunized to ASNase exhibited dynamic IgM, IgG, and IgE antibody responses. The severity of ASNase-induced anaphylaxis was found to be correlated with levels of IgG and IgE, but not IgM. Basophils from immunized mice were able to recognize and activate in response to ASNase ex vivo, and the extent of recognition and activation also correlated with the severity of anaphylaxis observed. Using a multivariable model that included all biomarkers significantly associated with anaphylaxis, independent predictors of ASNase-induced hypersensitivity reactions were found to be ASNase IC levels and ASNase-specific binding to leukocytes or basophils. Consistent with our multivariable analysis, we found that basophil depletion significantly protected mice from ASNase-induced hypersensitivity reactions, supporting that basophils are essential and can be used as a predictive marker of ASNase-induced anaphylaxis.

**Conclusions:**

Our study demonstrates the need for using tools that can detect both IC- and IgE-mediated hypersensitivity reactions to mitigate the risk of ASNase-induced hypersensitivity reactions during treatment.

## Introduction

The bacterial enzyme L-asparaginase (ASNase) is a critical component of pediatric acute lymphoblastic leukemia (ALL) treatment protocols and has recently been suggested as a potential therapeutic for other malignancies with similar characteristics as leukemias (1–4). Although ASNase is an effective agent for treating ALL, its use may be limited in some patients due to the development of immunotoxicity, with as many as 75% of ALL patients experiencing hypersensitivity reactions that may involve the formation of neutralizing anti-ASNase IgG antibodies (5, 6). These antibodies can neutralize plasma ASNase drug levels and cause hypersensitivity reactions, including anaphylaxis, which increases the risk of leukemia relapse (3, 6–10). Therefore, identifying patients who are at high risk of developing ASNase immunotoxicity is crucial for improving the effectiveness of ASNase therapy and reducing the risk of relapse in pediatric ALL patients.

We previously demonstrated that IgE- and IgG-mediated pathways are involved in ASNase hypersensitivity, both of which are associated with the severity of anaphylaxis (11, 12). Based on these results, we have developed tools that can discern between IgE- and IgG-mediated ASNase hypersensitivity (11–14). Others have explored IgE-mediated basophil activation and measurement of anti-ASNase antibody levels of various antibody isotypes (15, 16). However, predictive biomarkers of ASNase hypersensitivity in patients with ALL remain poorly studied, and reliable biomarkers for predicting clinical ASNase hypersensitivity reactions are lacking. No study has utilized immune cell-based approaches that focus on the Fc receptors directly implicated in anaphylaxis, such as FcγRIII and FcεRI, which are expressed on leukocytes and basophils, respectively. Therefore, there is an urgent need to evaluate strategies that can investigate both primary pathways of ASNase-induced hypersensitivity and detect the interaction of ASNase with immune cells expressing the key receptors involved in anaphylaxis.

The purpose of this study was to use our murine model of ASNase hypersensitivity to investigate the correlation between various immune response biomarkers and the severity of ASNase anaphylaxis towards developing a predictive test for improving clinical ASNase therapy. Furthermore, we determined the dynamics of anti-ASNase antibody responses, the formation of ASNase immune complexes (ICs), and ASNase-specific recognition of leukocytes/basophils relative to the onset of ASNase-induced anaphylaxis. Our results indicate an antibody-dependent association, where the IgE pathway of anaphylaxis and ASNase-specific basophil recognition best explain drug-induced anaphylaxis under conditions with low levels of anti-ASNase IgG antibodies. In contrast, the levels of ASNase ICs and the recognition of ASNase by leukocytes strongly correlated with the severity of ASNase-induced anaphylaxis under conditions with high anti-ASNase IgG antibody levels. Altogether, our results emphasize the importance of monitoring biomarkers of both IC and IgE-mediated hypersensitivity reactions to effectively identify sensitized patients before the onset of hypersensitivity reactions during treatment with ASNase.

## Materials and methods

### Animals

Adult (8-week-old) female C57BL/6J mice were purchased from the Jackson Laboratory (Bar Harbor, ME). Mice were housed in conventional cages under standardized conditions with controlled temperature and humidity with 12 h light/dark cycles. Mice were allowed *ad libitum* standard mouse chow diet and water during the entire period of handling. All experiments were approved by the Institutional Animal Care and Use Committee (IACUC) of the University of Pittsburgh and conducted per NIH guidelines for the care and use of laboratory animals.

### ASNase immunization and assessment of hypersensitivity reaction protocol

*E. coli ASNase immunization:* Eight-week-old female C57BL/6J mice were immunized with intraperitoneal injections of 10 µg of *E. coli* ASNase (BioVendor Laboratory Medicine Inc., Candler, NC) formulated with 1 mg of aluminum hydroxide adjuvant (Imject Alum; Thermo Scientific, Rockford, IL) on days 0 and 14 of the treatment protocol to sensitize the mice to ASNase as described previously by our group (12, 14, 17, 18).

*E. coli ASNase challenge:* To define the timeline of when ASNase hypersensitivities are induced in immunized mice, mice were challenged with a 100 μg dose of *E. coli* ASNase in 150 µL of PBS administered intravenously on day 0, 7, 14, 15, 17, 19, 21, 23 or 24 of immunization. The development of hypothermia, which determines the onset and severity of hypersensitivity reactions, was evaluated by monitoring rectal temperatures with a digital thermometer (model BAT-12, Physitemp Instruments, Clifton, NJ, USA) after the intravenous challenge with ASNase at different time points. Rectal temperature measurements were performed immediately before (time 0) and at different time points for 2 hours following the ASNase challenge. Pre- and post-challenge blood samples were collected on days 0, 7, 14, 15, 17, 19, 21, 23, and 24 of treatment by cardiac puncture at the end of the experiment for measuring various parameters as shown in (**Supplementary Figure 1A**). The area under the temperature versus time curve was calculated using the trapezoidal rule. More severe hypersensitivity reactions are indicated by a more significant change in core body temperature and lower AUC values. Throughout our analysis, mice were either sacrificed before or after the onset of hypersensitivity to assess differences in the severity of anaphylaxis as described previously (11–13).

### Detection of anti-ASNase IgM, IgG, and IgE antibodies by ELISA

Plasma samples were used to determine anti-ASNase IgG, and IgE antibodies from ASNase-immunized mice at different time points using an ELISA-based method, as described previously (11–13). Plasma samples from naïve non-immunized mice were used as negative controls, and rabbit polyclonal anti-ASNase IgG and IgM antibodies were used as positive controls (Rockland, USA). Mouse horseradish peroxidase–conjugated secondary antibodies to whole mouse IgG (A4416, Sigma), IgM (AP128P, Sigma) and IgE (PA1-84764, Thermo Fisher) were used for detection at a 1:1000 dilution. The samples were diluted to 1:400 in blocking buffer (1% dry milk powder in water) for IgG, 1:1000 for IgM, and 100 for IgE detection.

### Analysis of anti-ASNase IgG immune complex (IC) levels

ASNase ICs levels were measured in plasma collected 2 hr after the ASNase challenge as we described previously (11). Twenty-five µL of plasma was precipitated with an equal volume of 8% PEG (Sigma-Aldrich) and incubated with 6-8 µm diameter protein G-coated polystyrene particles (Spherotech, USA) for 60 min at 4°C on a shaker. After incubation, the FITC-labeled goat anti-mouse IgG Fc antibody (31547, Thermo Scientific) was incubated for 30 minutes at 4°C with protein-G beads, washed, and resuspended in 250 µL of running buffer (Miltenyi Biotech, Germany) for estimation of ASNase ICs by flow cytometry. Naïve mouse plasma and protein G beads alone were used as negative controls.

### ASNase drug activity determination

The ASNase drug activity in plasma samples was determined by monitoring the enzymatically coupled oxidation of reduced nicotinamide adenine dinucleotide (NADH) to NAD^+^ in a 96-well format, as described previously (13, 17).

### Quantification of plasma mouse mast cell protease-1 by ELISA

Mouse mast cell protease-1 (mMCP-1, a marker of mast cell degranulation) levels were determined from plasma samples using the Ready-SET-Go commercial ELISA kit according to the manufacturer’s instructions (Thermo Scientific, USA).

### ASNase-specific binding to total leukocytes (CD45^+^) and basophils (IgE^+^CD49b^+^)

No lyse/no-wash whole blood staining was performed using 25 µL of fresh blood samples collected by cardiac puncture after sacrificing the mice at different time points (**Supplementary Figure 1A**). Blood samples were stained with fluorochrome-labeled ASNase (12) and anti-mouse CD45 (clone REA737, VioGreen, 130-110-665, Miltenyi), CD49b (clone DX5, Alexa Fluor 488, 108913, Biolegend), and IgE (clone RME-1, PE, 406908, Biolegend) basophil markers for 1 hr at 4°C. After incubation, blood samples were suspended in a total of 2 mL of 1% BSA in PBS, and the samples were immediately analyzed using a MACSQuant Analyzer 10 (Miltenyi Biotec, Germany), as previously described (11, 12). The gating strategy is shown in **Supplementary Figure 1B**.

### Measuring basophilic activation using the basophil activation test

As we described previously, the basophil activation test (BAT) was performed (11, 12). Briefly, EDTA-containing whole blood was incubated for 15 minutes at 37°C. Blood samples were stimulated with *E. coli* ASNase at 0.1 IU/mL and incubated for 2 hours at 37°C in 5% CO_2_ to assess ASNase-mediated basophil activation. EM-95 (IgE-mediated positive control, 300 ng/mL), 2.4G2 (IgG-mediated positive control, 300 ng/mL), or RPMI medium alone served as positive and negative controls, respectively. After incubation, the reaction was quenched by adding 20 mM EDTA and incubated on ice for 10-15 minutes. Blood cells were blocked using 15% horse serum prepared in PBS for 30 minutes on ice, washed, and stained with anti-mouse IgE (clone RME-1, PE, 406908, Biolegend), CD49b (clone DX5, Alexa Fluor 488, 108913, Biolegend), CD200R3 (clone REA128, VioBlue, 130-102-748, Miltenyi), and CD200R1 mAbs (clone OX110, APC, 17-5201-82, eBiosciences) for 1 hr at 4°C. The blood samples were then lysed with RBCs lysis solution, washed with 1% BSA prepared in PBS, and samples were then analyzed on a flow cytometer. Basophils were detected based on the forward- and side-scatter characteristics and dual positive of IgE^+^ and CD49b^+^ staining. Up and down-regulation of CD200R1/CD200R3 on basophils was determined using a threshold that was defined by the fluorescence of unstimulated cells (negative control) and Fluorescence Minus One (FMO) controls. The percent change in CD200R1 or CD200R3 expression was calculated as the mean experimental expression minus the mean expression of the sample stimulated with medium alone, divided by the mean expression of the sample stimulated with medium alone.

### *In vivo* depletion of basophils (CD200R3^+^ leukocytes)

For the *in vivo* depletion of basophils (CD200R3^+^ leukocytes), mice were intravenously (i.v.) injected with anti-CD200R3 mAb (clone Ba103; 50 μg/mouse from Hycult Biotech, Netherlands) as previously described (19–22) one day before the ASNase challenge (i.e., Day 23). Basophil depletion was confirmed by flow cytometry.

### Statistical analysis

All statistical analysis was performed with R statistical software (version 2.13.2), Stata 16, and GraphPad Prism 8.4.3 (GraphPad Software, California, USA). Statistical analysis was performed with a two-tailed Student’s t-test between two groups and a one-way analysis of variance (ANOVA) for multiple groups, followed by Tukey’s *post hoc* test. The univariable analyses were conducted using simple linear regression to assess the association between rectal temperature AUC with anti-ASNase antibodies, basophil activation markers, and ASNase-specific recognition of leukocytes/basophils. Statistically significant factors from the univariable model were included in the multivariable linear regression model. Data are presented as mean ± standard deviation. A P value of less than 0.05 was considered significant and denoted as *P < 0.05; **P < 0.01; ***P < 0.001; ****P < 0.0001. Data are shown in mean ± SD.

## Results

### ASNase immunization leads to a dynamic anti-ASNase IgM, IgG, and IgE response

**Figure 1:**
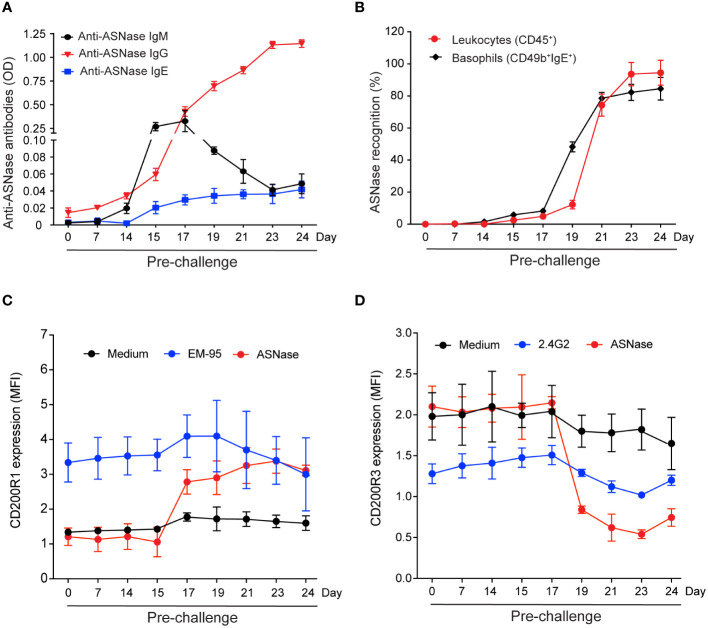
Levels of ASNase-specific antibodies and the magnitude of immune cell antigen-specific recognition and basophil activation vary based on the timing of the ASNase challenge. **(A)** Anti-ASNase IgM, IgG, and IgE antibody levels differed at the time of sacrifice after day 14. **(B)** ASNase binding to total leukocytes (CD45^+^) and basophils was higher at later time points. **(C)** IgE-mediated basophil activation was assessed by measuring the % increase of CD200R1 expression, and **(D)** IgG-mediated basophil activation was assessed by estimating the % decrease of CD200R3 expression on basophil after ASNase stimulation, n = 5 mice per time point. Data are shown in mean ± SD.

To determine which ASNase-specific immune response biomarker is best associated with the severity of ASNase hypersensitivity reactions, we varied the timing of the ASNase challenge after initial immunization or antigen exposure. The rationale for this design was to create conditions with varying levels and types of anti-ASNase antibodies and to assess when the presence of antibodies was associated with immune cell binding, activation, and the development of anaphylaxis. ASNase immunized mice demonstrate an initial elevated peak of anti-ASNase IgM before detecting anti-ASNase IgG, which later decreases and leads to a rise in anti-ASNase IgG on day 15 of the protocol and onwards, as expected from isotype class switching (**Figure 1A**). ASNase IgG levels increased more than 15-fold from their initial detection on day 15 (P < 0.001) to their peak levels on day 24 (P < 0.0001). We also measured anti-ASNase IgE, and the dynamics of anti-ASNase IgE were similar to IgG with a rise on day 15 of the protocol, which increased to statistically significant levels on day 17 (P < 0.001) and continued to gradually increase through day 24 of the experiment (**Figure 1A**, P < 0.001). Based on the dynamics of the various anti-ASNase antibody isotypes detected and their level, we next wanted to determine their role in antigen-specific recognition and their correlation with the severity of ASNase-induced hypersensitivity reactions upon antigen challenge.

### Immune cell antigen-specific recognition is detected after the onset of anti-ASNase antibodies and precedes IgE and IgG-mediated basophil activation

ASNase-specific recognition by leukocytes (i.e., CD45^+^ASNase^+^ cells) and basophils (i.e., CD49b^+^ IgE^+^ASNase^+^ cells) occurs two days after detection of anti-ASNase IgG antibodies (day 17, **Figures 1A, B**, P < 0.05). Two days after initial detection of ASNase-specific recognition (day 19), the percent of ASNase positive leukocytes and basophils rapidly increases (**Figure 1B**, P < 0.001), suggesting that ASNase ICs are forming at this time point due to the increased and higher levels of anti-ASNase IgG antibodies (11, 14). To determine if the anti-ASNase IgE levels detected (**Figure 1A**) are sufficient to lead to degranulation (14), we performed the basophil activation test (BAT) at all time points of our analysis using clinically relevant concentrations of ASNase (i.e., 0.1 IU/mL, **Figures 1C, D**) (23). Although we initially detected anti-ASNase IgE on day 17 of our protocol (**Figure 1A**, P < 0.001), we first detected IgE-mediated basophil activation (i.e., an increase in basophil CD200R1 expression) two days after initially detecting anti-ASNase IgE (day 19, **Figure 1C**, P < 0.001). These BAT results are consistent with the > 5-fold increase in basophil binding we detected between days 17 and 19 of our study (**Figure 1B**).

**Figure 2:**
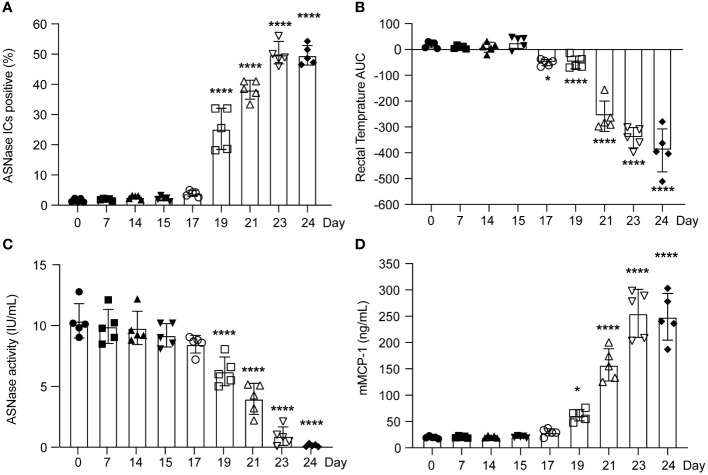
The severity of murine ASNase-induced hypersensitivity reactions varied according to the time between ASNase immunization and drug challenge. **(A)** ASNase IC levels increased around day 19. **(B)** The rectal temperatures of ASNase immunized mice were monitored for 2 hours after the challenge, and mice developed more severe hypothermia at later time points (i.e., lower rectal temperature AUC values). **(C)** ASNase drug levels were higher at earlier time points and were partially neutralized after day 17. **(D)** mMCP-1 levels increased at later time points. In our analysis, * and **** denote statistical significance compared with day 0, corresponding to P < 0.05 and 0.0001, respectively. Data are presented as mean ± SD, with n = 5 mice per group.

To determine the timepoint at which ASNase ICs form and are sufficient to activate basophils, we measured their *ex vivo* formation after adding clinically relevant concentrations of ASNase (i.e., 0.1 IU/mL) and measured the expression of basophil CD200R3 as a marker of IC-mediated basophil activation (20). Interestingly, ASNase ICs are first detected on day 19 (P < 0.0001, **Figure 2A**), which is four days after the initial detection of anti-ASNase IgG (day 15), and the levels of IC plateau by days 23-24 (P < 0.0001), similar to the anti-ASNase IgG profile (**Figure 1A**). Consistent with high antibody titers required for IC formation, anti-ASNase IgG levels were > 12-fold higher on day 19 relative to their initial detection (**Figure 1A**). Nevertheless, basophil activation associated with ASNase ICs was detected 2 days after the initial detection of ASNase ICs (day 21, **Figures 1D**, **2A**). While this suggests that the detection of ASNase ICs cannot be a direct indicator of ASNase-induced hypersensitivity reactions, the results are consistent with our leukocyte binding data, which demonstrate a > 4-fold increase in ASNase binding to leukocytes on day 21 versus day 19 (P < 0.0001, **Figure 1B**). Altogether, our results suggest that high antibody titers are required to form ASNase ICs capable of binding to and activating immune cells expressing Fcγ receptors that recognize IgG antibodies, such as basophils.

### The onset and severity of ASNase anaphylaxis varies depending on antibody responses, antigen-specific recognition, and basophil activation

Consistent with low to no detectable anti-ASNase IgE or IgG antibodies before day 15 of our experimental protocol (**Figure 1A**), we found that mice did not develop anaphylaxis on days 0 - 15, even after an initial ASNase exposure (**Figure 2B**). Instead, similar to the basophil and leukocyte binding results (**Figure 1B**), we observed that anaphylaxis is induced on day 17, and that the severity of hypersensitivity reactions increases through day 24 (**Figure 2B**). Interestingly, ASNase anaphylaxis induced on day 17 was associated with our initial detection of anti-ASNase IgE (**Figure 1A**) and CD49b^+^IgE^+^ASNase^+^ basophils (**Figure 1B**), but not with the detection of ASNase ICs (**Figure 2A**) or basophil activation (**Figures 1C, D**). These results suggest that hypersensitivity reactions on day 17 were most likely induced via the classical IgE pathway of anaphylaxis and not ASNase ICs. In contrast, immune complex-mediated anaphylaxis most likely contributed to the severity of these reactions on day 21 and onward, which is when we observed the initial detection of ICs (**Figure 2A**), a nearly 8-fold increase in CD45^+^ASNase^+^ cells (**Figure 1B**), IC-mediated basophil activation (**Figure 1D**), and a corresponding ≥ 3.5-fold increase in the severity of anaphylaxis relative to day 17 or 19 (**Figure 1A**). In agreement with our hypothesis that IC-mediated anaphylaxis is driving the rise in anaphylaxis severity from day 21 to 24 (**Figure 2B**), our data show that the formation of ASNase ICs follows a similar trend, where their levels increase from day 21-23 but plateau thereafter (**Figure 2A**). Likewise, free ASNase not associated with ICs is detectable through day 21 but is no longer measurable on days 23 and 24 (**Figure 2C**). Studies suggest that the detection of ICs alone is not sufficient to induce FcγR-dependent mast cell/basophil degranulation (11), and our results are consistent with these past studies, where we detect ASNase ICs on day 19 but their effect on ASNase-induced anaphylaxis (**Figure 2A**), ASNase binding to leukocytes (**Figure 1B**), and basophil activation (**Figure 1D**) is not apparent. Furthermore, measurement of the mast cell degranulation marker mMCP-1 supports our conclusions (**Figure 2D**), where it is modestly increased on day 19, likely due to IgE-mediated ASNase anaphylaxis, but sharply increases thereafter with the increased formation of ASNase ICs. Taken together, our results support that antigen-specific IgE antibodies mediate ASNase-induced anaphylaxis on days 17 and 19 and that ASNase ICs contribute to the severity of the reaction thereafter.

### Biomarkers of immune complex- and anti-ASNase IgE-induced anaphylaxis are independently associated with the severity of ASNase-induced hypersensitivity

**Figure 3:**
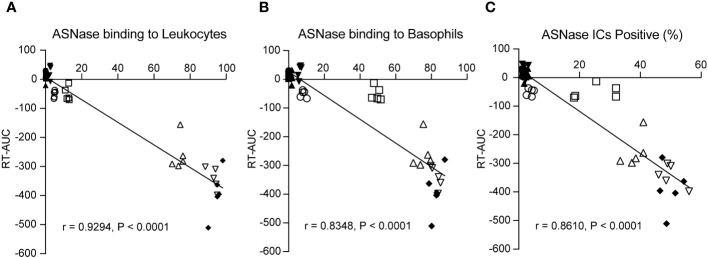
ASNase-specific basophil recognition and activation correlate with the onset and severity of ASNase-induced hypersensitivity. The severity of ASNase-induced hypersensitivity reactions (i.e., rectal temperature AUC) was positively associated with **(A)** ASNase recognition by leukocytes (CD45^+^), **(B)** ASNase recognition by basophils (CD49b^+^IgE^+^), and **(C)** increased ASNase IC levels. Each panel displays individual data points for each mouse studied.

Combining our various methods for detecting ASNase-specific anaphylaxis enables us to identify biomarkers that are modulated during anaphylaxis and to elucidate the mechanism of the adverse drug reaction. However, the data generated by our various methods are correlated. Therefore, we determined which biomarkers measured are independently associated with the severity of ASNase-induced anaphylaxis to identify those that may be more suitable for clinical implementation. To identify biomarkers that may be predictive of ASNase-induced anaphylaxis, we performed a multiple linear regression analysis using rectal temperature AUC (RT-AUC), which quantifies the severity of anaphylaxis, as the dependent variable and the ASNase-specific immune response data generated during this study as the independent variable. In the univariable analysis, anti-ASNase IgG/IgE levels, CD200R1/CD200R3 expression, ASNase leukocyte binding (i.e., quantity of ASNase bound CD45^+^ cells), ASNase basophil binding, and ASNase ICs significantly correlated with RT-AUC (**Table 1**). In the multivariable model that included all significant variables, ASNase binding to leukocytes, ASNase binding to basophil, and ASNase IC levels remained significant independent predictors of RT-AUC (**Figures 3A-C**, **Table 2**). The multivariable model accounted for 93% of the variation in RT-AUC (r^2 =^ 0.9267), indicating that these predictive biomarkers can capture much of the effect of the independent biological pathways leading to anaphylaxis in mice (**Figures 3A-C**, **Table 2**).

**Table 1:** Univariable associations between RT-AUC and predictor variables.

Variable	β coefficient (95% CI)	P-value	R-squared
Anti-ASNase IgM	437.9362 (-42.6704, 918.5427)	0.0731	0.0654
Anti-ASNase IgG	-244.3182 (-332.2949, -156.3416)	<0.0001	0.3938
Anti-ASNase IgE	-8549.863 (-10941.21, -6158.514)	<0.0001	0.5184
Expression of CD200R1	-17.78115 (-22.08308, -13.47922)	<0.0001	0.5900
Expression of CD200R3	81.92369 (60.6344, 103.213)	<0.0001	0.5900
ASNase binding to CD45	-3.337227 (-4.230372, -2.444082)	<0.0001	0.5404
ASNase binding to basophils	-3.749992 (-4.775423, -2.724561)	<0.0001	0.5297
ASNase ICs	-7.682266 (-8.55127, -6.813261)	<0.0001	0.8681

**Table 2:** Multivariable analysis between RT-AUC and significant univariable predictors.

Variable	β coefficient (95% CI)	P-value
Anti-ASNase IgG	12.12909 (-152.3671, 176.6253)	0.882
Anti-ASNase IgE	-1576.419 (-3995.371, 842.5316)	0.196
Expression of CD200R1	-5.511597 (-11.81009, 0.7868924)	0.085
Expression of CD200R3	-9.99627 (-41.85617, 21.86363)	0.530
ASNase binding to CD45	-3.181497 (-4.624936, -1.738058)	<0.001
ASNase binding to basophils	4.773264 (2.429193, 7.117335)	<0.001
ASNase ICs	-7.916645 (-9.954251, -5.87904)	<0.001

### Depletion of CD200R3^+^ cells protects from ASNase-induced hypersensitivity reactions in immunized mice

**Figure 4:**
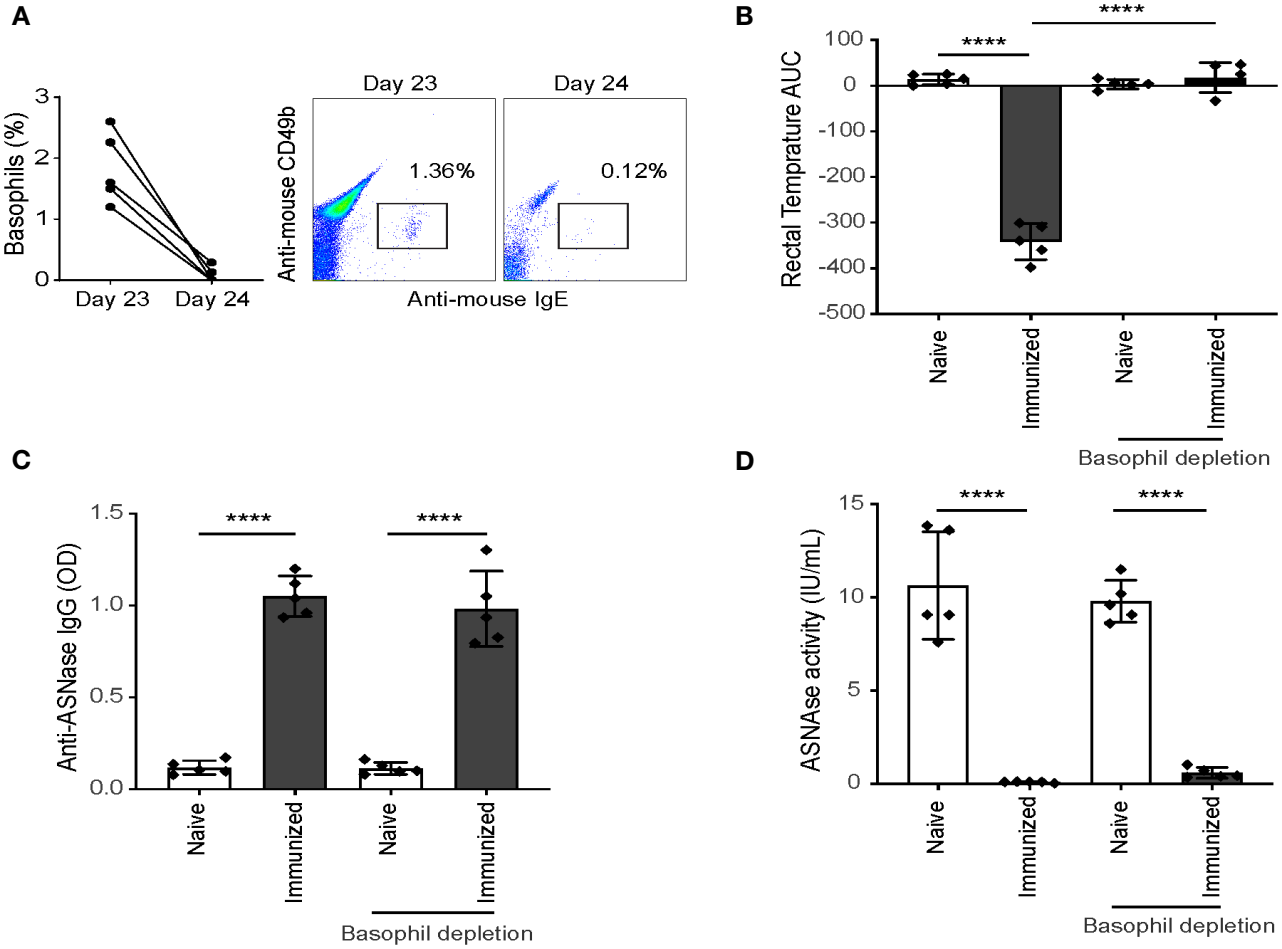
Depletion of CD200R3^+^ cells after ASNase sensitization protects mice from hypersensitivity. **(A)** Basophil depletion was confirmed by flow cytometry, with less than 0.05% of basophils detected in the blood after CD200R3^+^ cell depletion. Data shown as percentage of basophils from CD45^+^ leukocytes. **(B)** The rectal temperature of ASNase immunized mice were monitored for two hours after the ASNase challenge, and **(C)** anti-ASNase IgG, and **(D)** ASNase drug activity were measured after the ASNase challenge. n = 5 mice per group. Data are shown in mean ± SD. **** denotes P < 0.0001.

Previous studies (11, 12, 14) and the results from our independent association analysis suggest that basophils may not only be a biomarker of anaphylaxis but rather that they may be an effector cell leading to the onset of anaphylaxis. Next, we investigated the effector role of basophils in mice by depleting basophils using anti-CD200R3 mAb after ASNase immunization to ensure the mice had mounted an immune response before the antigen challenge, similar to previous studies (12, 19–21). Anti-CD200R3 mAb was administered intravenously on day 23 to deplete basophils one day before inducing anaphylaxis (**Supplementary Figure 2**), and less than 0.05% of basophils were detected in the blood after depletion with anti-CD200R3 (**Figure 4A**). Upon the ASNase challenge, the depleted basophil group was strongly protected from the development of anaphylaxis (**Figure 4B**). Furthermore, and as expected, we found that all immunized mice had similar anti-ASNase IgG levels despite the basophil depletion (**Figure 4C**) and that ASNase drug levels were not rescued by the basophil depletion (**Figure 4D**), consistent with the formation of ASNase ICs and lack of crosslinking or recognition by basophils. Taken together, the results from our basophil depletion studies suggest that basophils may contribute to the onset of ASNase-induced anaphylaxis and play an effector role in the phenotype, emphasizing the importance of monitoring these immune cells for assessing the risk of ASNase hypersensitivity in patients (20).

## Discussion

ASNase plays a crucial role as a component of multi-agent chemotherapy for the treatment of acute lymphoblastic leukemia. However, its effectiveness is often limited due to the development of an antigen-specific immune response. Currently, there is a lack of biomarkers available for the prediction of ASNase-induced immunotoxicity. In this study, we focused on identifying potential biomarkers that are highly correlated with the severity of ASNase hypersensitivity reactions using a preclinical murine model of ASNase hypersensitivity. Additionally, we investigated the immune cells responsible for the onset of anaphylaxis to better understand the mechanism behind this adverse drug reaction. Our findings indicate that there is a dynamic anti-ASNase antibody isotype response. However, detecting ASNase-specific antibodies alone is insufficient to predict hypersensitivity reactions. Rather, we show that high concentrations of anti-ASNase IgG antibodies are required to form ASNase-IgG ICs, which positively correlate with drug neutralization and ASNase binding to leukocytes. Although none of the methods tested showed a high correlation with mild anaphylaxis, our findings suggest that methods capable of detecting IgE- and IC-mediated anaphylaxis, such as BAT, are strongly linked to the severity of ASNase-induced hypersensitivity reactions. Additionally, our multivariable analysis revealed that ASNase-specific binding to leukocytes or basophils, as well as the concentrations of ASNase ICs, were all significant and independent predictors of the severity of ASNase-induced hypersensitivity reactions. Together, our analysis emphasizes that there are two main mechanisms of ASNase-induced hypersensitivity reactions and that sensitive methods detecting each mechanism is critical for predicting the onset of anaphylaxis.

In our preclinical model, we observe anaphylaxis as early as day 17 of the protocol (**Figure 2B**). This occurs prior to the detection of IC formation (**Figure 2A**) and basophil activation (**Figures 1C, D**). However, on the same day, we observe statistical increases in antigen-specific IgE and antigen-specific binding to basophils (**Figures 1A, B**). These results indicate a weak correlation between several of our measured biomarkers and the onset of mild ASNase-hypersensitivity reactions emphasizing the need for orthogonal strategies. Due to our past mechanistic studies and the lack of detectable ASNase ICs at this timepoint (11, 12), we posit that mild hypersensitivity reactions to ASNase are due to low levels of cell-associated anti-ASNase IgE. This conclusion is supported by the detection of IgE antibodies on day 17 (**Figure 1A**), binding of ASNase to basophils (**Figure 1B**), and IgE-mediated basophil activation on day 19 (**Figure 1C**).

We observed a dynamic anti-ASNase antibody response (**Figure 1A**), where antigen-specific IgM and IgG were first detected on day 15 of our protocol, followed by IgE on day 17. Notably, we found that anti-ASNase IgM antibodies did not affect drug levels (**Figure 2C**) and were not correlated with the onset of ASNase-induced anaphylaxis (**Figure 2B**). Anti-ASNase IgG antibodies have the potential to cause anaphylaxis when the antibody titers are high enough to lead to the formation of ICs, which then bind to the FcγRIII receptor of immune cells such as basophils and mast cells (24). Our findings align with this mechanism, as we observed that anti-ASNase IgG antibody levels peaked on day 24, which coincided with the peak concentrations of ICs, IC-mediated basophil activation, mMCP-1 release, and the highest severity of anaphylaxis.

Previous clinical studies have shown that single biomarker analysis of ASNase-induced immune responses only partially explains hypersensitivity reactions (15, 16). We therefore designed our study to identify novel biomarkers for predicting ASNase-induced hypersensitivity reactions. Based on the two primary mechanisms of ASNase-induced hypersensitivity reactions, we hypothesized that strategies assessing IgE- or immune complex-mediated anaphylaxis would best explain variability in the severity of hypersensitivity reactions. We therefore tested our various biomarkers for association with RT-AUC (**Tables 1**, **2**). Our analysis revealed that ASNase binding to leukocytes, ASNase binding to basophils, and ASNase IC levels remained significant independent predictors of RT-AUC (**Table 2**). Notably, the multivariable model accounted for 93% of the variation in the severity of ASNase-induced hypersensitivity reactions. These results are consistent with our hypothesis, where the binding of ASNase to leukocytes and basophils can be attributed to cell-associated IgE or IC binding to the FcγRIII of cells. Our results underscore the importance of developing immunoassays that can accurately detect antigen-specific recognition by immune cells expressing receptors associated with hypersensitivity reactions. Moreover, our preclinical findings may have clinical relevance to ASNase-induced hypersensitivity reactions, given that human basophils express both the high-affinity IgE receptor FcϵR1, and the stimulatory IgG receptor FcγRIII.

The cumulative evidence from our previous findings and the results of this study strongly suggest a significant involvement of basophils in ASNase-induced hypersensitivity reactions. To further investigate this hypothesis, we depleted CD200R3-positive basophils, which is a surface receptor expressed by these cells that is frequently targeted for basophil depletion (19, 25, 26). Because basophil activation and degranulation are downstream of antigen sensitization, we hypothesized that basophil depletion would not affect anti-ASNase antibody levels or drug neutralization, but rather protect from the severity of hypersensitivities. As predicted, basophil depletion had no discernable effect on antibody or ASNase plasma drug levels (**Figures 4C, D**) but led to a strong protection against the severity of ASNase-induced anaphylaxis (**Figure 4B**). The observed results align with assays showing the binding of ASNase to basophils and subsequent basophil activation (**Figures 1B-D**). These findings substantiate the potential importance of monitoring basophils as a predictive marker for ASNase-induced hypersensitivity reactions. However, since basophil depletion did not affect drug levels, these results highlight the need for novel prevention strategies to effectively mitigate immunotoxicity by reducing the development of anti-drug antibodies.

Our study indicates that a combination of strategies to monitor IgE- and IC-mediated hypersensitivity reactions would be the most effective in predicting ASNase-induced anaphylaxis. However, the clinical translation of our preclinical study is subject to limitations, such as the use of a mouse model, and thus requires clinical validation. Nonetheless, previous research suggests that the receptors and mechanisms involved in murine and human anaphylaxis are similar (24). Strong clinical evidence also supports the possibility of IC-mediated hypersensitivity reactions induced by ASNase (27). However, one limitation in assessing this pathway of anaphylaxis using human basophils is the lack of biomarkers available to assess IC-mediated basophil activation. Additionally, although anti-CD200R3 mAbs have been extensively used in basophil depletion studies (19, 25, 26) and research has confirmed their efficacy in depleting basophils but not CD200R3 positive mast cells (28), our results indicate a potential decrease in CD45^+^ leukocytes after basophil depletion and ASNase treatment (**Figure 4A**). Despite our previous findings that ASNase increases basophil frequencies and does not impact other leukocytes using this protocol (12), it remains uncertain whether anti-CD200R3 mAbs affected mast cell-mediated degranulation by ASNase in our analysis. Therefore, future studies will comprehensively assess the respective effects of anti-CD200R3 mAb and ASNase on mast cells and other leukocytes to verify our findings.

## Conclusion

In summary, we evaluated several potential predictive biomarkers of hypersensitivity reactions induced by ASNase using a preclinical murine model of immunotoxicity. In our study, univariable analysis identified several markers that correlated with the severity of anaphylaxis. Our multivariable analysis demonstrated that leukocyte/basophil binding to ASNase and ASNase ICs were independent predictors of hypersensitivity reactions. This highlights the significance of monitoring multiple pathways of anaphylaxis, emphasizing the need to assess various mechanisms in predicting and understanding the condition. Furthermore, our results suggest that basophils may not only serve as biomarkers of ASNase-induced hypersensitivity reactions, but also play a role as effector cells contributing to the severity of drug-induced anaphylaxis.

## Data availability statement

The original contributions presented in the study are included in the article/**Supplementary Material**. Further inquiries can be directed to the corresponding author.

## Ethics statement

The animal study was approved by Institutional Animal Care and Use Committee (IACUC) of the University of Pittsburgh. The study was conducted in accordance with the local legislation and institutional requirements.

## Author contributions

SR: Conceptualization, Data curation, Formal analysis, Writing – original draft, Writing – review & editing. KH: Formal analysis, Writing – original draft, Writing – review & editing. YZ: Data curation, Writing – review & editing. MR: Data curation, Writing – review & editing. CF: Conceptualization, Formal analysis, Funding acquisition, Writing – review & editing.
