# Supplementary material for: Asparaginase-specific basophil recognition and activation predict Asparaginase hypersensitivity in mice
Source: Front Immunol. 2024 Apr 15;15:1392099. doi: 10.3389/fimmu.2024.1392099 (PMC11057047; doi:10.3389/fimmu.2024.1392099)

SUPPLEMENTARY MATERIAL

Asparaginase-Specific Basophil Recognition and Activation Predict Asparaginase Hypersensitivity in Mice

Sanjay Rathod,[1] Keito Hoshitsuki,[1] Yin Zhu,[1] Manda Ramsey,[1] and Christian A. Fernandez[1]

[1]Center for Pharmacogenetics and Department of Pharmaceutical Sciences, University of Pittsburgh, Pittsburgh, PA 15261, USA

Running Title:

Asparaginase recognition and basophil activation predict asparaginase hypersensitivity.

Corresponding Author:
Christian A. Fernandez
Center for Pharmacogenetics
Department of Pharmaceutical Sciences
School of Pharmacy
335 Sutherland Drive
Pittsburgh, PA 15261
Phone: (412) 383-8108
chf63@pitt.edu

SUPPLEMENTAL FIGURE LEGENDS

Supplement Figure. 1. ASNase antibodies, antigen-specific recognition, and basophil activation were assessed as biomarkers for predicting the onset and severity of ASNase hypersensitivity in mice. (A) Schematic representation of the experimental design. Pre-ASNase challenge blood samples were used for measuring 1) anti-ASNase antibodies, 2) ASNase-specific binding to total leukocytes/basophils, and basophil activation. The severity of ASNase-induced hypersensitivity, 2) ASNase drug levels, 3) ASNase-immune complex levels, and 4) mMCP-1 concentrations were measured at the indicated time points. (B) Gating strategy for immune cells: $CD45^+$ defined total leukocytes and $CD45^+CD49b^+IgE^+$ defined basophils. ASNase-specific recognition and binding to $CD45^+$ and $CD49b^+IgE^+$ cells were identified by first gating leukocytes by scatter (FSC-A *vs* SSC-A). We isolated singlets based on FSC-A vs FSC-H, and then selected for CD45+ cells (leukocytes). Subsequently, we refined the population to identify ASNase-positive leukocytes (CD45+ASNase+) or basophils (CD49b+IgE+ASNase+). Gates were set using unstained and Fluorescence Minus One (FMO) controls for each blood sample marker.

Supplement Figure 2. Schematic of anti-CD200R3 challenge after ASNase immunization. After ASNase immunization, mice were pre-treated one day before the ASNase challenge with anti-CD200R3 or vehicle as schematically shown.

Supplement Figure 1

A.

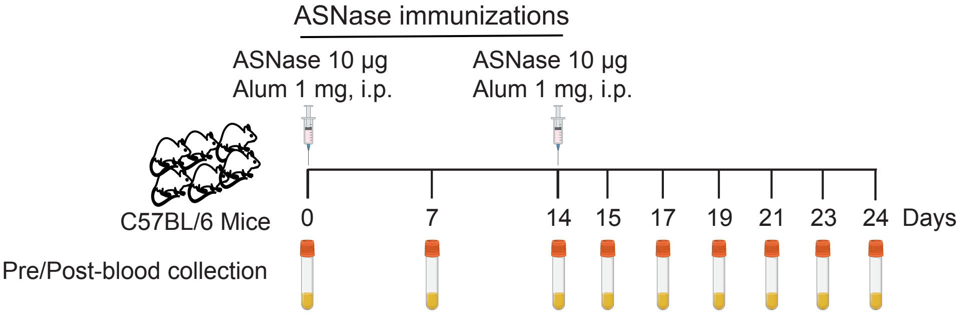

B.

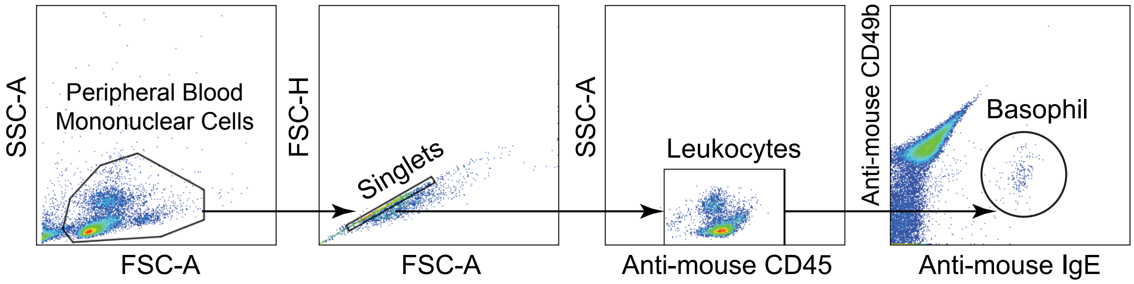

Supplement Figure 2

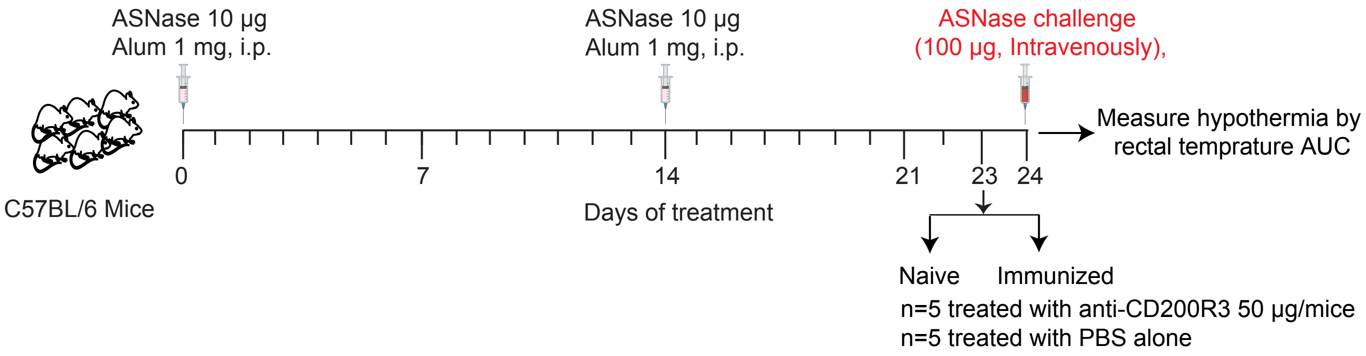

Supplement: Supplementary file 1 [file DataSheet_1.pdf]
